# Cellular Angiolipoma in the Breast: A Case Report and Literature Review of Its Pathogenesis

**DOI:** 10.7759/cureus.95090

**Published:** 2025-10-21

**Authors:** Xiaoming Fan, Qihui "Jim" Zhai, Jehan Abdulsattar

**Affiliations:** 1 Department of Pathology and Translational Pathobiology, Louisiana State University Health Shreveport, Shreveport, USA

**Keywords:** angiolipoma, breast, cellular angiolipoma, ultrasound, vascular neoplasm of breast

## Abstract

Angiolipoma is a benign variant of lipoma characterized by vascular proliferation among mature adipocytes, most commonly found on the extremities of young males. Cellular angiolipoma of the breast is extremely rare, with fewer than five cases reported in the English literature. We present a case of cellular angiolipoma in a 72-year-old female, manifesting as a 0.7 × 0.7 × 0.4 cm hyperechoic mass on ultrasound. Histopathological examination revealed a predominantly cellular lesion (~90%) interspersed with mature adipose tissue and abundant fibrin thrombi within small capillaries. The cellular component comprised spindle cells and vascular clusters with bland nuclei and low mitotic activity. Periodic acid-Schiff (PAS) staining and human herpesvirus 8 (HHV8) immunohistochemistry were negative. A diagnosis of benign cellular angiolipoma of the breast was made, with the tumor superficially involving the subcutaneous layer. The patient recovered uneventfully post-procedure. This report also discusses the pathophysiology and possible mechanisms underlying the development of angiolipoma.

## Introduction

Angiolipoma is a benign variant of lipoma characterized by vascular proliferation among mature adipocytes. While it is commonly found on the extremities of males in their second or third decade of life [1], it is a very rare lesion in the breast [2]. To the best of our knowledge, fewer than five cases have been reported in the English literature to date [3]. Although the diagnosis of angiolipoma is usually straightforward, it can be challenging in certain situations, such as in small core biopsies or when an angiolipoma presents as a morphological variant with predominant vascular components, also known as cellular angiolipoma. The diagnosis of cellular angiolipoma requires careful exclusion of its mimickers, including angiosarcoma, Kaposi sarcoma, angiomyolipoma, and myofibroblastoma of the breast. This distinction relies on a thorough correlation of clinical, imaging, and histomorphological findings. An appropriate immunohistochemical (IHC) panel is essential for accurate diagnosis, and in rare cases, molecular analysis may be required to definitively exclude these differentials. Here, we present a case of cellular angiolipoma of the breast in a 72-year-old female. We also review the literature on this rare but significant entity and discuss the pathogenesis of angiolipoma in detail.

## Case presentation

**Figure 1:**
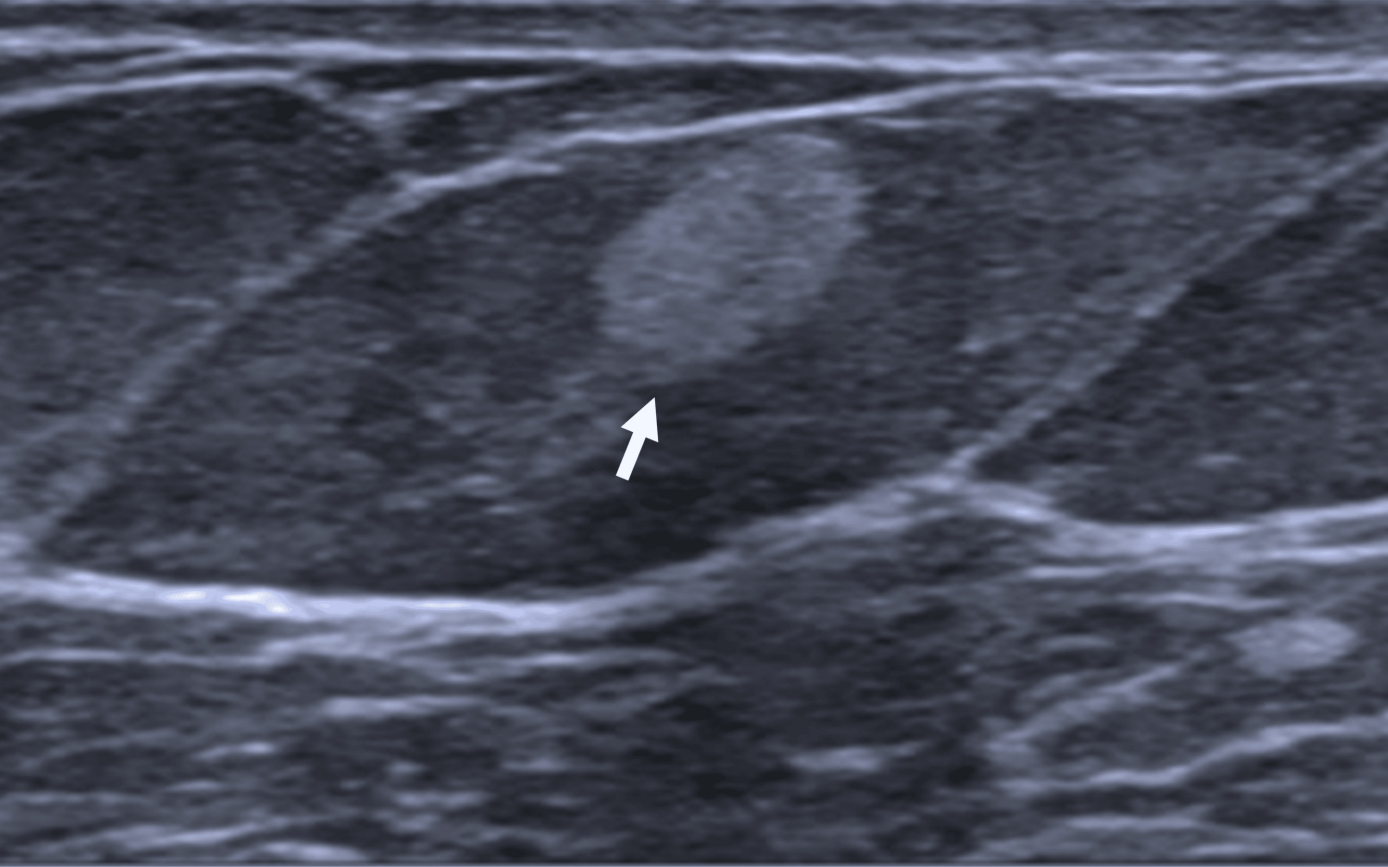
Ultrasound image showing the mass (white arrow) as a 0.7 × 0.7 × 0.4 cm hyperechoic lesion.

A 72-year-old female with a history of asthma, hypertension, obstructive sleep apnea, and allergic rhinitis presented for a digital screening mammogram. A superficial 0.7 cm mass was detected in the upper outer quadrant of the left breast. Subsequent ultrasound examination of the left breast revealed a 0.7 × 0.7 × 0.4 cm hyperechoic mass (Figure 1).

**Figure 2:**
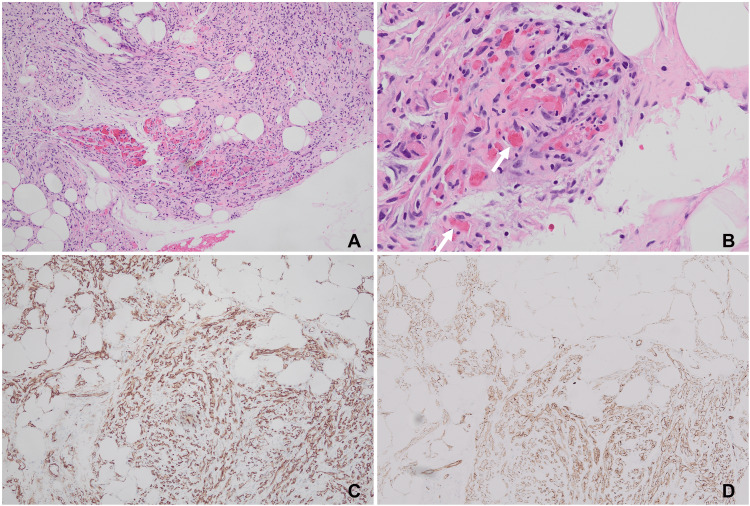
Histological examination of the lesion. (A) Histological examination reveals the mass as a predominantly cellular lesion with a minimal amount of mature adipose tissue. (B) Intravascular fibrin thrombi (arrow) are observed at the periphery of the lesion. (C) CD34 staining highlights vessels and a subset of spindle cells. (D) CD31 staining distinctly marks the vascular structures.

An ultrasound-guided vacuum-assisted core biopsy was performed, and a 2.7 × 1.6 × 0.7 cm aggregate of yellowish-tan soft tissue was sent to pathology for evaluation. Histopathological examination showed that the mass was a cellular lesion (~90%) mixed with a small amount of mature adipose tissue (~10%, Figure 2A).

Fibrin thrombi were abundant in the smaller capillaries (Figure 2B). The cellular part consisted mainly of spindle cells and clusters of vessels, as highlighted by CD34 and CD31 staining (Figure 2C-2D). The tumor cells had bland, inconspicuous nuclei with a low mitotic figure and a Ki-67 index of about 1%. Further, periodic acid-Schiff (PAS) stain and human herpesvirus 8 (HHV8) IHC stain were negative. No evidence of breast parenchyma was noted in the background. Taken together, the bland cellular morphology, low mitotic activity, positive IHC staining for vascular markers (CD34 and CD31), and negative staining for HHV8 help to rule out mimickers, including angiosarcoma, Kaposi sarcoma, angiomyolipoma, and myofibroblastoma of the breast. Based on the presence of all the characteristic components, such as mature adipose tissue, spindle cells, vascular clusters, and classic fibrin thrombi, a diagnosis of benign cellular angiolipoma superficially involving the subcutaneous breast tissue was rendered. The patient had an uneventful recovery after the procedure.

## Discussion

Cellular angiolipoma is a variant of angiolipoma characterized by predominantly cellular angiomatous tissue within the lesion. This entity was first formally introduced by Hunt et al. in 1990 [4]. In the available literature, cellular angiolipoma predominantly occurs in the trunk and extremities [5]. There are only a few case reports and studies of cellular angiolipoma of the breast [3,6-8]. Cellular angiolipoma of the breast has an average age of presentation of 64 years and an average size of 7 mm [6]. In our case, the patient was 72 years old, and the size of the lesion was 7 mm, consistent with previous studies.

Histopathologically, cellular angiolipoma presents with a predominantly cellular lesion interspersed with a small amount of adipose tissue. While the precise proportion of cellularity is not clearly defined, a ratio of approximately 90% cellular components to 10% adipose tissue is generally acceptable for diagnosing cellular angiolipoma [5]. Key diagnostic features include a cellular area with spindle cells, an increased number of capillary vessels, and mature adipose tissue. A crucial diagnostic clue is the presence of intravascular fibrin thrombi. Cellular angiolipoma may sometimes exhibit focal cellular atypia, pleomorphism, a few mitotic figures, and scattered apoptotic bodies [6].

Given these features, careful examination is essential to rule out malignant mimickers such as angiosarcoma and Kaposi sarcoma. Recognizing characteristics of these malignant tumors, such as prominent cellular atypia, high mitotic figures, irregular blood vessels, and blood extravasation, is crucial for accurate diagnosis. IHC stains like CD34, CD31, and HHV8 are helpful in differentiating these conditions. When spindle cells are more prominent, cellular angiolipoma may also be mistaken for myofibroblastoma of the breast [7]. In such cases, positive staining for vascular markers like CD31, the absence of significant cellular atypia, and the presence of peripheral fibrin thrombi are key features that support a diagnosis of cellular angiolipoma.

In the current case, the inconspicuous appearance of spindle cells and blood vessels, typical intravascular fibrin thrombi, low mitotic figures and a low Ki-67 index, and negative HHV8 stain confirmed the diagnosis of cellular angiolipoma.

Given the benign nature of angiolipoma, research on its pathogenesis and genetics is limited. Older studies indicated that most angiolipomas have a normal karyotype [9,10]. However, recent molecular studies have identified some abnormalities. For example, Panagopoulos et al. [11] reported the loss or structural rearrangement of chromosome 13 in three angiolipoma cases.

In a study using whole-exome sequencing and targeted ultra-deep sequencing [12], the authors reported that 80% of angiolipoma cases harbored mutations in the protein kinase D2 (PRKD2) gene. PRKD2 plays a crucial role in endothelial cell proliferation, migration, and angiogenesis through the vascular endothelial growth factor receptor-2 and fibroblast growth factor receptor-1 pathways [13]. Additionally, PRKD2 can bind directly to phosphoinositide 3-kinase (PI3K) and promote phosphoinositide 3-kinase/protein kinase B (PI3K/Akt) pathway-induced angiogenesis and tumorigenesis [14,15]. Furthermore, a study by Aaggini et al. [16] identified gain-of-function mutations in phosphatidylinositol-4,5-bisphosphate 3-kinase catalytic subunit alpha (PIK3CA), a key component of the PI3K pathway, in 70% of conventional angiolipomas (9 of 13 cases) and in all cellular angiolipomas (5 cases). These studies highlight the role of PI3K pathway-induced angiogenesis in the pathogenesis of angiolipoma. However, further research is needed to confirm this concept.

Taken together, we present a rare case of cellular angiolipoma of the breast. A thorough investigation should be conducted to make an accurate diagnosis, especially to carefully rule out its malignant mimickers, including angiosarcoma and Kaposi sarcoma. Significant advances have been made in understanding the pathogenesis and molecular aspects of this entity in recent years; however, more studies are warranted to better understand angiolipoma.

## Conclusions

In the present report, we describe a rare case of cellular angiolipoma occurring in the breast, an atypical location for this entity. Particular attention was given to excluding morphologic mimickers, including angiosarcoma, Kaposi sarcoma, angiomyolipoma, and mammary myofibroblastoma. In addition, we conducted a comprehensive review of the literature to contextualize this uncommon but clinically relevant diagnosis and to provide an in-depth discussion of the proposed pathogenesis of angiolipoma.
